# Assessment of intracranial vessels in association with carotid atherosclerosis and brain vascular lesions in rheumatoid arthritis

**DOI:** 10.1186/s13075-017-1422-x

**Published:** 2017-09-26

**Authors:** Csaba Oláh, Zsófia Kardos, Mariann Sepsi, Attila Sas, László Kostyál, Harjit Pal Bhattoa, Katalin Hodosi, György Kerekes, László Tamási, Attila Valikovics, Dániel Bereczki, Zoltán Szekanecz

**Affiliations:** 1 Department of Neurosurgery, Borsod County Teaching Hospital, Miskolc, Hungary; 2 Department of Rheumatology, Borsod County Teaching Hospital, Miskolc, Hungary; 3 Department of Radiology, Borsod County Teaching Hospital, Miskolc, Hungary; 4 Department of Neurology, Borsod County Teaching Hospital, Miskolc, Hungary; 5 Department of Laboratory Medicine, Faculty of Medicine, University of Debrecen, Debrecen, Hungary; 6 Division of Rheumatology, Department of Medicine, Faculty of Medicine, University of Debrecen, 98 Nagyerdei Street, H-4032 Debrecen, Hungary; 7 Department of Angiology, University of Debrecen Faculty of Medicine, Debrecen, Hungary; 8 Department of Neurology, University of Debrecen Faculty of Medicine, Debrecen, Hungary; 9 Department of Neurology, Semmelweis University, Budapest, Hungary

**Keywords:** Rheumatoid arthritis, Cerebrovascular disease, Stroke, Transcranial Doppler, Carotid artery, Methotrexate, Biologic therapy

## Abstract

**Background:**

Stroke has been associated with rheumatoid arthritis (RA). We assessed patients with RA and healthy control subjects by transcranial Doppler (TCD), carotid ultrasonography and brain magnetic resonance imaging (MRI).

**Methods:**

Altogether, 41 female patients with RA undergoing methotrexate (MTX) or biologic treatment and 60 age-matched control subjects underwent TCD assessment of the middle cerebral artery (MCA) and basilar artery. Pulsatility index (PI), resistivity (resistance) index (RI) and circulatory reserve capacity (CRC) were determined at rest (r) and after apnoea (a) and hyperventilation (h). The presence of carotid plaques and carotid intima-media thickness (cIMT) were also determined. Intracerebral vascular lesions were investigated by brain MRI.

**Results:**

MCA PI and RI values at rest and after apnoea were significantly increased in the total and MTX-treated RA populations vs control subjects. MCA CRC was also impaired, and basilar artery PI was higher in RA. More patients with RA had carotid plaques and increased cIMT. Linear regression analysis revealed that left PI(r) and RI(r) correlated with disease duration and that left PI(r), RI(r), PI(a), PI(h) and basilar PI correlated with disease activity. Right CRC inversely correlated with 28-joint Disease Activity Score. Disease activity was an independent determinant of left PI(a) and right CRC. Compared with long-term MTX treatment alone, the use of biologics in combination with MTX was associated with less impaired cerebral circulation. Impaired cerebral circulation was also associated with measures of carotid atherosclerosis.

**Conclusions:**

To our knowledge, this is the first study to show increased distal MCA and basilar artery occlusion in RA as determined by TCD. Patients with RA also had CRC defects. We also confirmed increased carotid plaque formation and increased cIMT. Biologics may beneficially influence some parameters in the intracranial vessels.

## Background

Rheumatoid arthritis (RA) is a chronic inflammatory rheumatic disease often involving the vascular and nervous systems [1]. Accelerated atherosclerosis and increased stroke morbidity and mortality have been associated with RA [2–5]. According to a recent meta-analysis, there is an excess risk of stroke (ORs ranging from 1.51 to 2.13) [5]. Yet, to date, no systematic evaluation of intracranial circulation has been performed.

Transcranial Doppler (TCD), first described in 1982, is a non-invasive ultrasound (US) technique used to measure cerebral blood flow velocity in the major intracranial arteries. It involves use of low-frequency (<2 MHz) US waves to assess cerebral arteries through relatively thin bone windows. TCD allows dynamic monitoring of cerebral circulation with a high temporal resolution. It is relatively inexpensive, reproducible and portable [6, 7]. TCD may provide important information about the pathophysiology and prognosis of cerebrovascular ischemia [7]. When assessing the middle cerebral artery (MCA) by TCD, mean flow velocity (MFV), pulsatility index (PI) and the PI/MFV ratio are prognostic factors for recurrent vascular events [8]. Furthermore, asymptomatic MCA stenosis, which can be detected by TCD, may be an important risk for stroke [9].

One limitation of TCD is that approximately 8–20% of individuals have inadequate temporal acoustic windows (TAWs) [10]. There is an increased prevalence of TAW failure in females and in elderly people [10]. Very recently, we assessed TAW failure in patients with RA compared with control subjects and found that TAW failure was significantly more common among patients with RA (Z. Kardos, C. Oláh, M. Sepsi, A. Sas, L. Kostyál, T. Bóta, H. Bhattoa, K. Hodosi, G. Kerekes, L. Tamási, D. Bereczki, Z. Szekanecz, unpublished data, September 2017).

As described above, patients with RA are at very high risk for cardiovascular and cerebrovascular disease. Numerous studies have been performed on cardiovascular disease and its surrogate markers. However, because stroke is the second most common vascular complication in RA, there is a great need to assess cerebrovascular risk as well. Because very little information is available with respect to intracranial vessels in RA, we sought to assess the following: (1) to determine circulation in the MCA, as well as in basilar arteries, by TCD to determine whether there are abnormalities in intracranial circulation in RA; (2) to assess the effects of anti-tumour necrosis factor α biologics vs methotrexate (MTX) treatment on TCD parameters; and (3) to correlate intracranial circulation with carotid atherosclerosis and brain vascular lesions. Therefore, we performed an extensive study using TCD in patients with RA undergoing MTX or biologic treatment and compared them with healthy control subjects. The functional TCD assessment included the determination of several functional parameters described later. We also assessed carotid atherosclerosis by US and performed brain magnetic resonance imaging (MRI) to visualise vascular lesions as well as signs of emollition and atrophy. To our knowledge, this is the first study to assess intracranial vessels in patients with RA by TCD in conjunction with carotid and brain examinations.

## Methods

### Patients and control subjects

Altogether, 41 patients with RA undergoing regular follow-up at the Semmelweis Teaching Hospital, Miskolc, Hungary, were recruited for the study. All these patients had accessible TAWs as determined before the TCD investigations. None of the patients with RA and control subjects had any previous vascular events. The major characteristics of these 41 patients are shown in Table 1. All patients were female, and their mean age was 58.2 ± 9.7 years. Their mean disease duration was 12.2 ± 8.0 years. Altogether, 70% were immunoglobulin M (IgM) rheumatoid factor (RF)-positive, and 65% were anti-cyclic citrullinated peptide 2 antibody (anti-CCP2)-positive. Among these patients, 12 were biologic-free. They had been receiving MTX for a mean of 6.2 ± 5.8 years in an average dose of 14.6 ± 6.1 mg/week. Altogether, 29 patients had been receiving biologics (15 infliximab [IFX] and 14 tocilizumab [TCZ]) as first-line biologic treatment, in combination with MTX, for a mean duration of 5.3 ± 2.0 years. The characteristics of the MTX- and biologic-treated RA subsets are shown in Table 1.

**Table 1:** General characteristics and laboratory markers of assessed patients with rheumatoid arthritis and control subjects

	RA total	RA MTX	RA biologic	Control subjects
No. of subjects	41	12	29	60
Age, years	58.24 ± 9.72	58.67 ± 9.88	58.07 ± 9.88	58.42 ± 4.41
Disease duration. years	12.15 ± 8.03	11.00 ± 8.63	12.62 ± 7.87	–
RF positivity, %	70%	82%	66%	–
Anti-CCP positivity, %	65%	75%	64%	–
MTX duration, years	6.15 ± 5.82	5.83 ± 4.57	6.28 ± 3.54	–
MTX dose, mg/week	14.63 ± 6.11	14.17 ± 5.15	14.83 ± 6.54	–
Biologic duration, years	5.31 ± 2.04	–	5.31 ± 2.04	–
DAS28	2.44 ± 0.87	2.88 ± 0.75	2.26 ± 0.86	–
ESR, mm/h	17.07 ± 15.09	17.00 ± 9.16	17.10 ± 17.10	5.30 ± 2.40
hsCRP, mg/L	3.93 ± 4.53	5.31 ± 3.90	3.36 ± 4.70	0.30 ± 0.30
BMI, kg/m^2^	28.38 ± 5.51	28.08 ± 6.77	28.51 ± 5.02	30.20 ± 8.70
TC, mmol/L	5.34 ± 1.08	5.63 ± 1.00	5.23 ± 1.10	5.27 ± 1.15
HDL-C, mmol/L	1.47 ± 0.43	1.38 ± 0.53	1.49 ± 0.40	1.70 ± 0.42
TC/HDL-C ratio	3.94 ± 1.36	4.70 ± 3.73	3.73 ± 1.20	3.62 ± 0.95
LDL-C, mmol/L	3.17 ± 0.82	3.36 ± 0.90	3.12 ± 0.81	3.40 ± 0.94
TG, mmol/L	1.46 ± 0.68	1.53 ± 0.76	1.43 ± 0.66	1.49 ± 0.61
Lp(a), ng/L	260.14 ± 317.20	345.29 ± 426.55	239.59 ± 290.74	–
ApoA/ApoB ratio	1.81 ± 0.51	1.71 ± 0.49	1.83 ± 0.53	–

*Abbreviations: Apo* Apolipoprotein, *BMI* Body mass index, *CCP* Cyclic citrullinated peptide, *CRC* Cerebrovascular reserve capacity, *DAS28* 28-joint Disease Activity Score, *ESR* Erythrocyte sedimentation rate, *HDL-C* High-density lipoprotein cholesterol, *hsCRP* High-sensitivity C-reactive protein, *LDL-C* Low-density lipoprotein cholesterol, *Lp(a)* Serum lipoprotein A, *MTX* Methotrexate, *RA* Rheumatoid arthritis, *RF* Rheumatoid factor, *TC* Total cholesterol, *TG* Triglyceride

For this study, a cohort of 60 age-matched women without RA who were undergoing TCD investigations were chosen as the control group. These individuals were part of a TCD screening program conducted in the Miskolc region. The mean age of these 60 individuals was 58.4 ± 4.4 years. The age of control subjects was not significantly different from that of patients with RA.

Ethical approval (1046-63/2015) was obtained from the University of Miskolc Regional/Institutional Review Board. All patients signed informed consent forms. The study was performed according to the Declaration of Helsinki.

### Determination of temporal acoustic windows

Before the TCD assessment was carried out by US, an ultrasonographer (MS) determined whether the right and left TAWs were detectable and available for TCD measurements. Only patients and control subjects with detectable TAWs could be further analysed by TCD.

### Transcranial Doppler assessment

The Doppler effect means that if US waves strike a moving object, such as an erythrocyte, the reflected wave undergoes a change in frequency (Doppler shift) which is directly proportional to the velocity of the reflector. A US frequency of < 2 MHz is required to penetrate the skull and reach the intracranial vasculature. Acoustic windows are either foramina or thin bone that allow US waves to reach the cerebral circulation. The TAW located above the zygomatic ridge is most frequently used for detection of the anterior cerebral artery, MCA or posterior cerebral artery, as well as the terminal internal carotid artery (ICA) [6]. Unfortunately, as described above, approximately 8–20% of individuals [10] may have TAW failure.

In this study, MCAs were assessed by TCD using a Multi-Dop T Digital (DWL Compumedics GmbH, Singen, Germany) device. Among TCD indices, MFV (cm/second), peak systolic flow velocity (PSFV) and end diastolic flow velocity (EDFV) are key parameters in TCD assessment. MFV is the mean velocity within a cardiac cycle. Increased MFV may indicate stenosis, vasospasm or hyperdynamic flow. A decreased value may indicate hypotension, decreased cerebral blood flow and intracranial pressure.

The PI, calculated as (PSFV − EDFV)/MFV, provides information on downstream cerebral vascular resistance (normal range 0.6–1.2). Proximal stenosis or occlusion may lower the PI below 0.5, whereas distal occlusion or constriction may increase the PI above 1.2. The resistivity (resistance) index (RI), calculated as (PSFV − EDFV)/PSFV (normal range 0.4–0.7), indicates downstream resistance.

Cerebrovascular reserve capacity (CRC) is the physiological vasoconstrictive and vasodilatory function of precapillary (resistance) arteries that maintains optimal cerebral blood flow even if cerebral perfusion pressure changes. In the MCAs, CRC in percent is calculated as (PSFV − MFV)/MFV × 100. A normal response in MFV values after acetazolamide is a 29 cm/second increase in women and 21 cm/second increase in men [11], which corresponds to 49.3% and 38.9% CRC, respectively.

First, MFV, PI and RI were determined at rest (r) [MFV(r), PI(r), RI(r)]. Then, provocation manoeuvres were performed, including hyperventilation (h) for 30 seconds [MFV(h), PI(h), RI(h)], followed by, after normal breathing, apnoea (a) for 30 seconds [MFV(a), PI(a), RI(a)] [12]. After these manoeuvres, the flow velocity in MCA should increase by at least 15% under normal conditions; otherwise, severely impaired CRC is present [12].

The TCD procedure was performed by a single technician (MS) and validated by a neurosurgeon/neuroradiologist (CO). All the above-mentioned parameters were determined in the RA cohort. In control subjects, all parameters described above could also be assessed.

### Carotid artery ultrasound examination

Carotid artery assessments were carried out in all patients with RA (*n* = 41) and control subjects (*n* = 60) using a duplex US system (Vivid e; GE Healthcare, Wauwatosa, WI, USA) using a 5-MHz linear array transducer (GE 8 L-RS probe; GE Healthcare) as described by us elsewhere [13]. Assessments were performed by a single observer (AS). In brief, five measurements were performed on the posterior wall of the distal part of common carotid artery (CCA), proximal to the carotid bulb. Longitudinal high-resolution B-mode US scans were employed over both the right and left CCAs and were R-synchronised and recorded. Carotid intima-media thickness (cIMT) was defined as the distance between the first and second echogenic lines from the lumen, taking the average of five measurements on both sides. cIMT values were expressed in millimetres.

During US assessments, all visible plaques were detected and counted, and their characteristics (fibrous, calcified or soft, presence or absence of stenosis) were determined. Soft plaques representing unstable plaques are more dangerous than fibrous or calcified ones [13, 14]. A ‘plaque score’ of 0–5 was also assigned to each patient, with scores of 0–5 representing no plaque, fibrous, non-stenotic calcified, stenotic calcified, soft and stenotic soft, respectively.

### Brain MRI investigations

All 41 patients with RA also underwent brain MRI studies to be assessed for focal vascular lesions, emollition and atrophy. For this purpose, a MAGNETOM Verio 3 T MRI instrument (Siemens, Munich, Germany) was used. All MRI scans were obtained by a single radiologist (LK) and read by a neurosurgeon/neuroradiologist (CO). The presence or absence of vascular lesions, emollition and/or atrophy was noted.

### Laboratory assessments

Serum IgM RF and high-sensitivity C-reactive protein (hsCRP) were assessed by quantitative nephelometry (COBAS MIRA Plus; Roche Diagnostics, Indianapolis, IN, USA), using RF and CRP reagents, respectively (both from DIALAB, Wiener Neudorf, Austria). RF levels > 50 IU/ml indicated seropositivity, and hsCRP levels > 5 mg/L were considered elevated. Anti-CCP autoantibodies were detected in serum samples using the second-generation Immunoscan-RA CCP2 enzyme-linked immunosorbent assay (Euro-Diagnostica, Arnhem, The Netherlands). The assay was performed according to the instructions of the manufacturer. A concentration > 25 IU/ml indicated seropositivity.

After overnight fasting, blood samples were taken from the patients and control subjects for total cholesterol (TC), low-density lipoprotein cholesterol (LDL-C), high-density lipoprotein cholesterol (HDL-C) and triglycerides. Lipids were determined using routine laboratory methods. Serum lipoprotein A [Lp(a)] was assessed by latex-sensitised immunoturbidimetry (Roche Diagnostics). Serum apolipoprotein A (ApoA) and ApoB levels were measured by immunoturbidimetry using Tina-quant Apoliporotein A and Apoliporotein B reagents (Roche Diagnostics) and a cobas Integra 700 analyser (Roche Diagnostics). ApoB/ApoA ratio, a good marker for atherosclerotic risk [15, 16], was calculated (Table 1).

### Statistical analysis

The statistical analysis was performed with IBM SPSS Statistics version 22 software (IBM, Armonk, NY, USA). The data are expressed as mean ± SD and as frequency and percentage. Continuous variables were evaluated by paired two-tailed *t* tests and Wilcoxon’s test. Nominal variables were compared between groups using the chi-square or Fisher’s exact test, as appropriate. Simple correlations were determined by Spearman’s correlation analysis. Multiple linear regression using the stepwise method was used to determine correlations and independent associations between parameters. TCD/carotid parameters were the dependent variables, and other clinical and laboratory parameters (e.g., age, disease duration, RF and anti-CCP positivity, MTX dose and duration, biologic therapy duration, 28-joint Disease Activity Score [DAS28], erythrocyte sedimentation rate, hsCRP, body mass index [BMI], lipids) were independent variables. The β standardised linear coefficients showing linear correlations between two parameters were determined. The B regression coefficient (with 95% CI) indicated an independent association between the dependent and independent variables during changes. *p* values < 0.05 were considered significant.

## Results

### Comparative description of RA subsets

With respect to clinical and laboratory characteristics (Table 1), the total RA cohort, MTX- and biologic-treated patients did not differ from each other in most respects. However, MTX-treated patients had significantly higher CRP (5.3 ± 3.9 mg/L vs 3.4 ± 4.7 mg/L; *p* < 0.05) and mean DAS28 (2.88 ± 0.75 vs 2.26 ± 0.86; *p* = 0.006) than any biologic-treated ones (Table 1). Numerous lipid parameters have been studied. Results showing no major differences between RA subsets are provided in Table 1.

### TCD assessments in patients with RA, RA subsets and control subjects

**Fig. 1:**
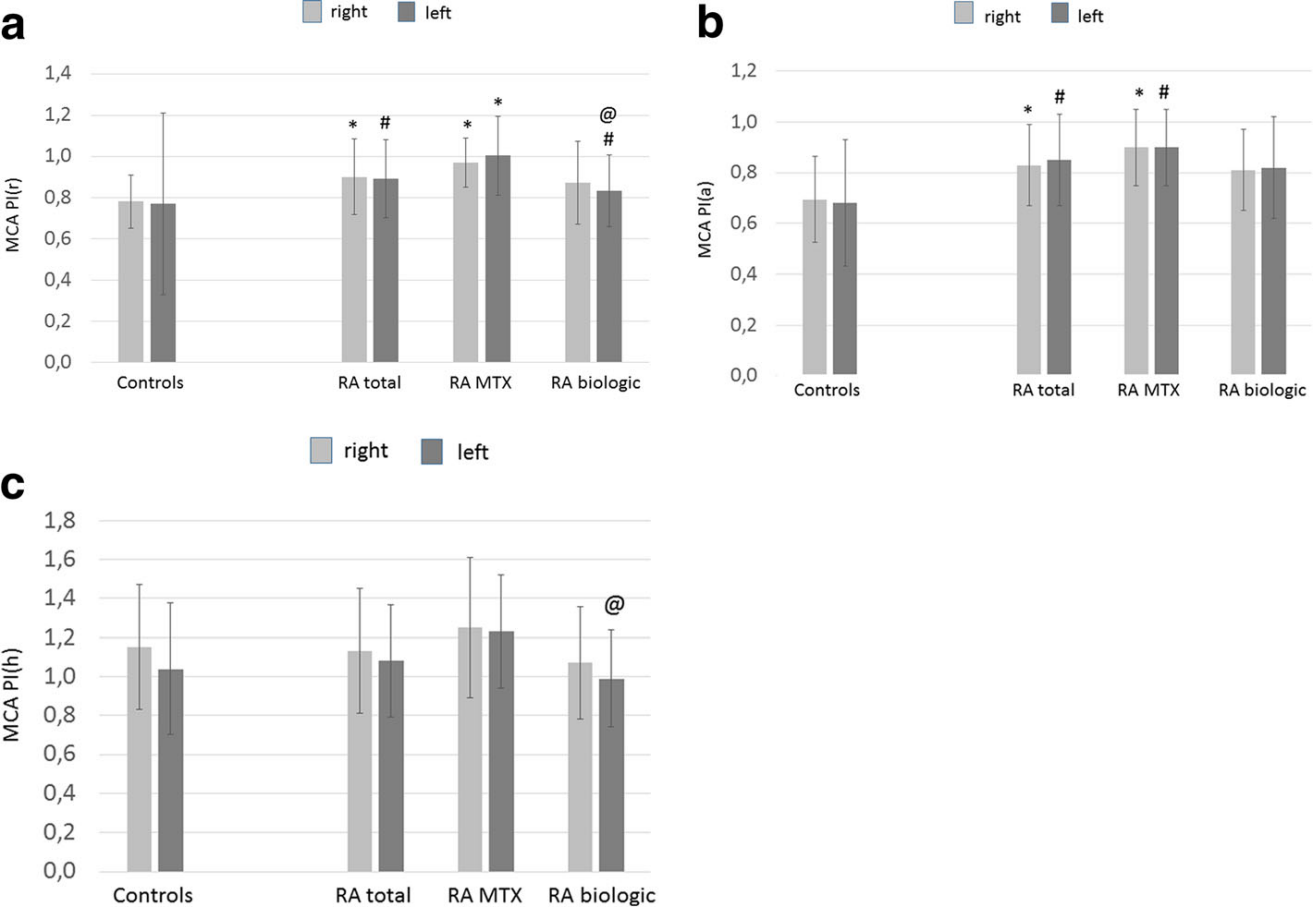
MCA PI(r) (**a**), PI(a) (**b**) and PI(h) (**c**) values in patients with RA and control subjects. *See text* for abbreviations. **a** *p < 0.001 vs controls; ^#^ *p* < 0.05 vs controls; ^@^ *p* < 0.001 vs MTX. **b** *p < 0.001 vs controls; ^#^ *p* < 0.05 vs controls. **c** ^@^ *p* < 0.05 vs MTX

**Fig. 2:**
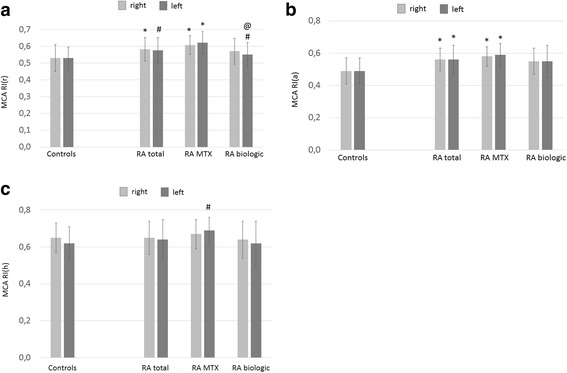
MCA RI(r) (**a**), RI(a) (**b**) and RI(h) (**c**) values in patients with RA and control subjects. *See text* for abbreviations. **a** *p < 0.001 vs controls; ^#^ *p* < 0.05 vs controls; ^@^ *p* < 0.05 vs MTX **b** *p < 0.001 vs controls **c** ^#^ *p* < 0.05 vs controls

Right and left MCA PI, RI and MFV values at rest (r) and after hyperventilation (h) and apnoea (a); right and left CRC values; and basilar artery PI(r) and MFV(r) values of patients with RA and control subjects have been evaluated and are presented. MCA PI and RI values detected at rest (r), after apnoea (a) and hyperventilation (h) are shown in Figs. 1 and 2. With respect to right and left MCA PI(r), all patients with RA (right 0.90 ± 0.18, *p* < 0.001; left 0.89 ± 0.19, *p* = 0.003) and MTX-treated patients (right 0.97 ± 0.12, *p* < 0.001; left 1.00 ± 0.19, *p* < 0.001) had significantly higher PI(r) values than control subjects (right 0.78 ± 0.13; left 0.77 ± 0.44) (Fig. 1a). Biologic-treated patients with RA exerted significantly higher left MCA PI(r) than control subjects (0.83 ± 0.17 vs 0.77 ± 0.44, *p* = 0.021) but significantly lower left MCA PI(r) than MTX-treated patients (0.83 ± 0.17 vs 1.00 ± 0.19, *p* = 0.021) (Fig. 1a).

With regard to right and left MCA PI(a), the total RA cohort (right 0.83 ± 0.16, *p* < 0.001; left 0.85 ± 0.03, *p* = 0.002) and MTX-treated patients (right 0.90 ± 0.15, *p* < 0.001; left 0.90 ± 0.15, *p* = 0.009) had significantly higher PI(a) values than control subjects (right 0.69 ± 0.17; left 0.68 ± 0.25) (Fig. 1b). On one hand, the total and MTX-treated RA population did not differ from control subjects in PI(h) values. On the other hand, biologic-treated patients with RA exerted significantly lower left MCA PI(h) than MTX-treated patients (0.99 ± 0.25 vs 1.23 ± 0.29, *p* = 0.029) (Fig. 1c).

Right and left MCA RI(r) values were also significantly higher in the total (right 0.58 ± 0.07, *p* < 0.001; left 0.58 ± 0.08, *p* = 0.004) and MTX-treated patients with RA (right 0.61 ± 0.06, *p* < 0.001; left 0.62 ± 0.07, *p* < 0.001) than control subjects (right 0.53 ± 0.08; left 0.53 ± 0.07) (Fig. 2a). Biologic-treated patients with RA had significantly higher left MCA RI(r) than control subjects (0.55 ± 0.07 vs 0.53 ± 0.07, *p* = 0.018) but significantly lower left MCA RI(r) than MTX-treated patients (0.55 ± 0.07 vs 0.62 ± 0.07, *p* = 0.018) (Fig. 2a).

**Fig. 3:**
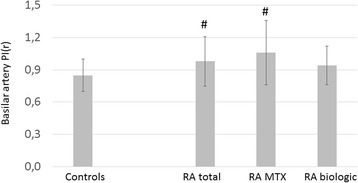
Basilar artery PI(r) values in patients with RA and control subjects. *See text* for abbreviations. ^#^ *p* < 0.05 vs controls

Right and left RI(a) was also significantly higher in all patients with RA (right 0.56 ± 0.07, *p* < 0.001; left 0.56 ± 0.02, *p* < 0.001) and in MTX-treated patients with RA (right 0.58 ± 0.06, *p* < 0.001; left 0.59 ± 0.07, *p* < 0.001) than in control subjects (right 0.49 ± 0.08; left 0.49 ± 0.07) (Fig. 2b). Finally, patients with RA did not differ from control subjects in right MCA RI(h) values. However, MTX-treated patients with RA exerted significantly higher left MCA RI(h) than control subjects (0.69 ± 0.07 vs 0.62 ± 0.29, *p* = 0.09) (Fig. 2c). Basilar artery PI(r) was also increased in the total RA cohort (0.98 ± 0.26, *p* = 0.005) and in MTX-treated patients (1.05 ± 0.38, *p* = 0.001) compared with control subjects (0.85 ± 0.15) (Fig. 3).

**Fig. 4:**
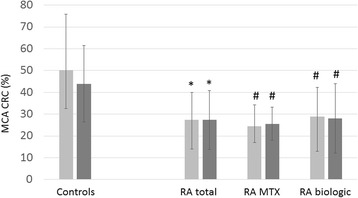
MCA CRC values in patients with RA and control subjects. *See text* for abbreviations. *p < 0.001 vs controls; ^#^ *p* < 0.05 vs controls

The right and left CRC was significantly impaired in all patients with RA (right 27.5 ± 12.5%, *p* < 0.001; left 27.3 ± 13.5, *p* < 0.001), MTX-treated patients with RA (right 24.5 ± 9.8, *p* = 0.005; left 25.6 ± 7.6, *p* = 0.002) and biologic-treated patients with RA (right 28.8 ± 13.5, *p* < 0.001; left 28.2 ± 15.9, *p* < 0.001) compared with control subjects (right 50.2 ± 30.6; left 44.0 ± 17.6) (Fig. 4).

Finally, in addition to the significant differences described above, right MCA PI(r), PI(a), PI(h), RI(r), RI(a), RI(h), left MCA PI(a), RI(a), RI(h), basilar artery PI(r) values exerted a tendency of being increased in MTX-treated vs biologic-treated patients (Figs. 1, 2 and 3). Also, right and left MCA CRC was non-significantly lower in MTX-treated than in biologic-treated patients (Fig. 4).

### Carotid artery assessments

The right and left carotid arteries could be assessed in all patients with RA (*n* = 41) and control subjects (*n* = 60). With respect to the right carotid, the percentage of patients with at least one plaque was significantly higher in all patients with RA (51%; *p* = 0.021) and MTX-treated subjects (67%; *p* = 0.016) than in control subjects (27%). There was no difference between the subsets with regard to left carotid plaque percentages (Table 2). Likewise, the right but not the left carotid plaque score as defined above was also significantly higher in the total (1.22 ± 1.35; *p* = 0.016) and MTX-treated RA subset (1.58 ± 1.31; *p* = 0.08) (Table 2).

**Table 2:** Carotid intima-media thickness and brain magnetic resonance imaging assessments

	RA total (*n* = 41)	RA MTX (*n* = 12)	RA biologic (*n* = 29)	Control subjects (*n* = 60)	*p* Value			
					RA total vs control subjects	RA MTX vs control subjects	RA biologic vs control subjects	RA MTX vs RA biologic
Carotid ultrasound studies								
Right cIMT (mm)	0.72 ± 0.24	0.72 ± 0.25	0.72 ± 0.23	0.40 ± 0.28	<0.001	<0.001	<0.001	
Left cIMT (mm)	0.73 ± 0.23	0.69 ± 0.22	0.75 ± 0.24	0.40 ± 0.28	<0.001	<0.001	<0.001	
Right carotid plaques (%)	21 (51%)	8 (67%)	13 (45%)	16 (27%)	0.021	0.016		
Left carotid plaques (%)	18 (44%)	6 (50%)	12 (41%)	21 (35%)				
Right carotid plaque score	1.22 ± 1.35	1.58 ± 1.31	1.07 ± 1.36	0.60 ± 0.99	0.016	0.008		
Left carotid plaque score	1.00 ± 1.16	1.25 ± 1.55	0.90 ± 0.98	0.78 ± 1.12				
Brain MRI studies								
Right vascular lesions (%)	16 (39%)	5 (42%)	11 (38%)					
Left vascular lesions (%)	16 (39%)	5 (42%)	11 (38%)					
Emollition	2 (5%)	0	2 (7%)					
Atrophy	8 (20%)	1 (8%)	7 (24%)					

*Abbreviations: cIMT* Carotid intima-media thickness, *MRI* Magnetic resonance imaging, *MTX* Methotrexate, *RA* Rheumatoid arthritis

The cIMT of the right carotid artery was 0.72 ± 0.24 mm in the total RA cohort and 0.40 ± 0.28 mm in control subjects (*p* < 0.001). Similarly, the left carotid cIMT was greater in RA (0.73 ± 0.23 mm) than in control subjects (0.40 ± 0.28 mm) (*p* < 0.001). Right and left cIMT were also significantly higher in MTX-treated (right 0.72 ± 0.25 mm, *p* < 0.001; left 0.69 ± 0.22 mm, *p* < 0.001) and biologic-treated patients (right 0.72 ± 0.23, *p* < 0.001; left 0.75 ± 0.24 mm0, *p* < 0.001) than in control subjects (right 0.40 ± 0.28 mm; left 0.40 ± 0.28 mm) (Table 2).

### Brain MRI investigations

As described above, 3-T brain MRI investigations were carried out in the 41 patients with RA. Altogether, 39% of all patients with RA, 42% of MTX- and 38% of biologic-treated patients with RA had at least one vascular lesion in the right and left cerebral hemispheres. Only two patients had signs of emollition, whereas eight patients had cerebral atrophy. There were no statistical differences between MTX- and biologic-treated patients (Table 2).

### Associations of TCD, carotid features and other parameters in patients with RA

As described above, multiple linear regression analysis was performed to determine associations between TCD/carotid parameters as dependent variables and other independent clinical and laboratory variables. As shown in Table 3, numerous TCD variables correlated with age. When assessing disease-related factors, left MCA PI(r) and PI(h) correlated positively, whereas right MCA CRC inversely correlated, with disease activity. With respect to metabolic factors, right PI(h) and right cIMT correlated with BMI. Left RI(h) inversely correlated with HDL-C.

**Table 3:** Associations between transcranial Doppler/carotid parameters and other variables^a^

Parameter 1	Parameter 2	B (95% CI)	β	*p* Value
Right PI(r)	Age	0.007 (0.002–0.013)	0.384	0.011
Right RI(r)	Age	0.004 (0.002–0.006)	0.508	0.001
Right PI(h)	BMI	0.024 (0.006–0.042)	0.410	0.011
Right RI(h)	Age	0.004 (0.002–0.007)	0.456	0.002
Left PI(r)	Age	0.009 (0.002–0.017)	0.447	0.012
Left RI(r)	Age	0.004 (0.001–0.007)	0.459	0.011
Left PI(a)	DAS28	0.086 (0.013–0.159)	0.368	0.023
Left PI(h)	CRP	0.028 (0.005–0.050)	0.442	0.016
	DAS28	0.087 (0.008–0.166)	0.372	0.032
Left RI(h)	HDL-C	−0.104 (−0.203–0.006)	−0.415	0.039
Basilar PI	Age	0.008 (0.002–0.014)	0.325	0.010
Right CRC	DAS28	−4.633 (−9.047–0.219)	−0.326	0.040
Right cIMT	BMI	0.002 (0–00.3)	0.306	0.015

*Abbreviations: β* Standardised linear coefficient, *B (+95% CI)* Regression coefficient, *BMI* Body mass index, *CRP* C-reactive protein, *CRC* Cerebrovascular reserve capacity, *DAS28* 28-joint Disease Activity Score, *HDL-C* High-density lipoprotein cholesterol, *PI* Pulsatility index, *RI* Resistivity (resistance) index

^a^Multiple linear regression

We also correlated TCD variables with carotid US and brain MRI results. When comparing patients with RA with (*n* = 18) and without (*n* = 23) left carotid plaques, we found that right MCA PI(a), RI(r) and RI(a), as well as left MCA PI(a), PI(h) and RI(a), values were significantly higher in patients with carotid plaques (Table 4). In addition, right MCA PI(h) significantly correlated with right cIMT (*R* = 0.377, *p* = 0.018) (data not shown).

**Table 4:** Associations between transcranial Doppler and left carotid artery plaques

TCD variable	Presence of plaque (*n* = 18)	Absence of plaque (*n* = 23)	*p* Value
Right PI(a)	0.92 ± 0.17	0.77 ± 0.12	0.002
Right RI(r)	0.61 ± 0.07	0.56 ± 0.07	0.021
Right RI(a)	0.59 ± 0.09	0.53 ± 0.05	0.007
Left PI(a)	0.94 ± 0.18	0.79 ± 0.17	0.032
Left PI(h)	1.23 ± 0.29	0.94 ± 0.25	0.021
Left RI(a)	0.61 ± 0.10	0.53 ± 0.07	0.022

*Abbreviations: PI* Pulsatility index, *RI* Resistivity (resistance) index, *TCD* Transcranial Doppler

TCD and carotid parameters did not show significant associations with any brain MRI findings (data not shown).

## Discussion

RA has been associated with accelerated atherosclerosis leading to increased cardiovascular and cerebrovascular morbidity and mortality [2–5, 17, 18]. Cerebrovascular disease and stroke may occur with uncontrolled, more severe disease [5, 17–19]. It is very important to assess the intracranial vessels as well as the carotid arteries in order to determine cerebrovascular risk and the preclinical status of patients [14, 19, 20]. To our knowledge, to date, no systematic evaluation of intracranial circulation using functional TCD has been performed. In addition, we believe this is the first study including use of TCD, assessment of carotid arteries and brain MRI in the very same RA cohort. Because of the lack of literature data, we could not compare our findings with data reported by others.

TCD allows dynamic monitoring of cerebral circulation [6]. TCD is a very convenient, cheap and portable technique that can be used at the bedside. TCD may be able to assess the pathophysiology and outcome of cerebral ischemia [7]. Moreover, MCA may be the most relevant vessel to study by TCD. MCA stenosis, as well as MCA PI, MFV and PI/MFV ratio, has been associated with stroke, and all are important predictors of future cerebrovascular events [8, 9].

TAW failure may limit the performance of TCD. Approximately 8–20% of people in the general population have undetectable TAW [10]. There has been no report on TAW failure in patients with RA. Very recently, we have found that TAW failure was significantly more common in RA than among the general population (Kardos et al., submitted). Therefore, before performing TCD measurements, we looked for right and left TAWs. Eventually, 41 consecutive patients were suitable for TCD, carotid artery and brain MRI studies.

We performed a detailed TCD assessment in a homogeneous RA population and with age-matched, healthy control subjects. We also compared patients treated with MTX and biologics including IFX and TCZ. The total, MTX-treated and biologic-treated patients did not differ from each other in most respects. MTX-treated patients had higher disease activity than biologic-treated patients.

With respect to MCA, PI and RI at rest and after apnoea measured on both sides were significantly higher in RA, indicating increased distal occlusion and resistance, respectively. Patients with RA also had an impaired CRC, as indicated by lower CRC. Significantly higher PI was detected in the basilar artery as well. With regard to treatment, MTX-treated patients had higher PI and RI values than biologic-treated patients. For example, left MCA PI(r), PI(h) and RI(r) were significantly lower in biologic-treated patients than in MTX-treated patients. Furthermore, right MCA PI(r) and RI(r), as well as right and left MCA PI(a) and RI(a), were still increased in MTX-treated patients compared with control subjects, whereas these parameters were similar to those of biologic-treated patients. These data suggest that patients with RA have impaired circulation in the MCA and basilar arteries compared with control subjects. Furthermore, biologics may improve numerous MCA parameters to a greater extent than MTX.

The carotid arteries were also assessed for signs of atherosclerosis. More patients with RA than control subjects had at least one plaque in the right carotid artery, and right carotid plaque score was also higher in patients with RA. There were no differences among RA subsets regarding the presence of plaques or plaque scores. The cIMT of the right and left carotid arteries was also much higher in the total, MTX-treated and biologic-treated patients with RA than in control subjects. We and others have previously reported increased cIMT and plaques in RA [13, 20–25]. In this cohort, the differences in cIMT between patients with RA and control subjects are really large, probably due to the fact that mostly patients with severe RA who needed biologics were selected. Carotid arteries have not yet been studied in conjunction with TCD in patients with RA.

Brain MRI investigations were carried out to determine cerebral vascular lesions. About half of patients with RA developed vascular lesions in the right or left hemisphere. Somewhat greater proportions of MTX-treated than biologic-treated patients who developed vascular lesions. We also detected signs of cerebral emollition and atrophy in about 5% and 24%, respectively, of patients with RA. We have not found any reports with respect to cerebral emollition in RA. Wartolowska et al. [26] also detected some atrophy in patients with RA. Bekkelund et al. [27] performed a quantitative cerebral MRI study in 1995. They did not find any differences in the prevalence of atrophy in patients with RA and control subjects [27].

Multiple linear regression analysis suggested the associations of multiple TCD parameters with age. Some parameters also correlated with RA disease activity. When TCD and carotid assessment results were correlated, numerous TCD variables were increased in patients with left carotid plaques. Right PI(h) also correlated with right cIMT. These correlations should be confirmed in larger future studies. However, comparative TCD, carotid US and brain MRI studies have not yet been performed in patients with RA.

## Conclusions

To our knowledge, this is the first detailed study using TCD to assess intracranial arteries in RA compared with control subjects. We also included carotid assessment and brain MRI in the same patients. We found more pronounced distal occlusion and resistance of the MCA along with impaired CRC in patients with RA compared with control subjects. We confirmed that carotid plaques and increased cIMT occur in patients with RA, and we described cerebral vascular lesions, emollition and atrophy in a group of patients with RA. With respect to therapy, biologic treatment, in comparison with MTX, may have more pronounced effects on some TCD PI and RI indices. Preclinical cerebrovascular screening may be important in determining stroke risk in individuals with RA.

## Key messages

- Increased distal MCA and basilar artery occlusion was detected in patients with RA, as determined by TCD.
- Patients with RA also have CRC defects.
- Biologics may beneficially influence some parameters in the intracranial vessels.

## Abbreviations

Apo: Apolipoprotein
BMI: Body mass index
CCA: Common carotid artery
CCP: Cyclic citrullinated peptide
cIMT: Carotid intima-media thickness
CRP: C-reactive protein
CRC: Cerebrovascular reserve capacity
DAS28: 28-joint Disease Activity Score
EDFV: End diastolic flow velocity
ESR: Erythrocyte sedimentation rate
HDL-C: High-density lipoprotein cholesterol
hsCRP: High-sensitivity C-reactive protein
IFX: Infliximab
IgM: Immunoglobulin M
LDL-C: Low-density lipoprotein cholesterol
Lp(a): Serum lipoprotein A
MCA: Middle cerebral artery
MFV: Mean flow velocity
MRI: Magnetic resonance imaging
MTX: Methotrexate
PI: Pulsatility index
PSFV: Peak systolic flow velocity
RA: Rheumatoid arthritis
RF: Rheumatoid factor
RI: Resistivity (resistance) index
TAW: Temporal acoustic window
TC: Total cholesterol
TCD: Transcranial Doppler
TCZ: Tocilizumab
TG: Triglyceride
US: Ultrasound
